# Feline Mammary Tumors: A Comprehensive Review of Histological Classification Schemes, Grading Systems, and Prognostic Factors

**DOI:** 10.3390/vetsci12080736

**Published:** 2025-08-05

**Authors:** Joana Rodrigues-Jesus, Hugo Vilhena, Ana Canadas-Sousa, Patrícia Dias-Pereira

**Affiliations:** 1 Department of Pathology and Molecular Immunology, School of Medicine and Biomedical Sciences, ICBAS-UP, University of Porto, 4050-313 Porto, Portugal; jjoanarodrigues@gmail.com (J.R.-J.); canadas.ana@gmail.com (A.C.-S.); 2 Associated Laboratory for Green Chemistry (LAQV), REQUIMTE, University of Porto, 4050-313 Porto, Portugal; 3 Department of Veterinary Clinics, School of Medicine and Biomedical Sciences, ICBAS-UP, University of Porto, 4050-313 Porto, Portugal; hcrvilhena@hotmail.com; 4 Animal and Veterinary Research Centre (CECAV), University of Trás-os-Montes e Alto Douro, 5000-801 Vila Real, Portugal; 5 Vasco da Gama Research Centre (CIVG), Department of Veterinary Sciences, Vasco da Gama University School (EUVG), 3020-210 Coimbra, Portugal; 6 Associate Laboratory of Animal and Veterinary Sciences AL4AnimaLS, 1300-477 Lisbon, Portugal

**Keywords:** feline mammary tumors, prognosis, histological classification, histological grading, molecular phenotyping, review

## Abstract

As the body of knowledge on feline mammary tumors (FMTs) continues to grow, their histological classification and grading systems have undergone revisions and updates to better reflect the biological behavior of these tumors. In this review, the historical evolution of these frameworks is traced and later revisited in the context of their prognostic relevance. Numerous studies have investigated clinicopathological prognostic factors in feline mammary carcinomas (FMCs); however, the heterogeneity in assessment methods, inclusion criteria for survival analysis, and the clinical endpoints considered can often complicate direct comparisons across different studies and may contribute to seemingly conflicting results. Furthermore, the small cohort size of many studies limits the robustness and transferability of their findings. This paper provides an updated overview of the epidemiological, clinical, and pathological prognostic factors of these tumors, while also highlighting current challenges, methodological limitations, and areas for future improvement.

## Simple Summary

Mammary tumors are among the most common neoplasms in cats and frequently exhibit aggressive behaviors. This review summarizes the historical evolution of the histological classification and grading systems for feline mammary carcinomas, highlighting recent revisions. Furthermore, an overview of epidemiological, clinical, and pathological prognostic factors reported in the literature, from earlier studies to more recent research and newly proposed markers, is provided. By bringing together current knowledge and discussing its limitations, we aim to facilitate and fuel future improvements in research and clinical practice on neoplastic diseases with impacts on feline species.

## 1. Introduction

Mammary gland tumors are consensually reported to be among the three most frequent neoplasms in feline species, representing between 11% and 35.5% of all feline tumors, depending on geographical location [1,2,3]. Even though mammary tumors predominantly affect queens, the disease has occasionally been described in male cats [4,5,6,7,8,9], representing between 0.5% and 5% of the feline mammary carcinoma (FMC) diagnoses [2,10,11,12,13] and exhibiting a biological behavior similar to that observed in queens [8]. Mammary neoplasms mostly affect middle-aged to older queens with a mean age between 10 and 12 years [2,14,15]. These tumors present as solitary or multiple nodules, which may be mobile in the subcutaneous tissue or attached to the underlying tissues of one or several mammary glands [16,17,18]. Typically, between 86 and 97% of the feline mammary lesions display a malignant biological behavior [2,19,20,21]; hence, it is recommended that every feline mammary lesion be treated as potentially malignant [17]. Indeed, due to the rapid progression of FMC, cats are frequently in an advanced stage of the disease at the time of diagnosis [16,22], and regional lymph node and distant metastasis, as well as lymphovascular invasion (LVI), might already be present [14,23,24,25]. Surgery alone is often the treatment of choice, with a median survival time of around 8–15 months [12,26,27]. Local recurrence and distant metastasis are common, and tumor dissemination mainly affects the regional lymph nodes and lungs; however, other organs can also be involved, viz., the thyroid glands, heart, central nervous system, liver, spleen, adrenal glands, kidneys, muscle, bone, and sciatic nerve [4,13,22,26,28,29,30]. Adjuvant therapies have been investigated; however, significant beneficial effects have yet to be demonstrated [26,28], warranting the need for a deeper understanding of FMCs.

Age, breed, and hormonal exposure have been recognized as risk factors for the development of feline mammary neoplasms [31]. Cats between 10 and 14 years of age are at increased risk of mammary tumor development [2,19,32]. Two studies suggested that Siamese cats have a higher incidence of FMC and develop the disease at a younger age [10,33]; however, the breed might have been overrepresented [31]. Early ovariohysterectomy has been documented as having a significant protective effect in cats, reducing the risk of FMC development by 91% and 86% in queens neutered before 6 months and 1 year of age, respectively [34]. Additionally, an increased risk of developing mammary lesions has been observed in cats exposed to exogenous progestogens, irrespective of gender [6,8,35,36].

Over the years, numerous potential prognostic markers for FMC have been studied, from subject-related parameters to clinical, histopathological, histochemical, immunohistochemical, genetic, hematological, and serological parameters [15,30,37,38,39,40,41,42,43,44]. In 2015, Zappulli and co-workers conducted a comprehensive review of the published literature on FMC and determined that tumor size, lymph node metastasis, LVI, and Elston and Ellis (EE) histological grading were the most dependable prognostic parameters [15]. Since then, a decade of additional research has expanded the body of knowledge on these tumors. This review begins with a historical overview of the evolution in the histological classification and histological grading of feline mammary tumors (FMTs). Following this, we seek to present an updated analysis of the prognostic factors in FMC, organized into two main categories: (1) epidemiological and clinical parameters, and (2) pathological features. Thus, this review aims to consolidate insights into the most readily accessible parameters for veterinary clinicians, with the goal of supporting decision-making in the management of FMC and facilitating future research.

## 2. Histological Classification and Grading Systems

### 2.1. Histological Classification

Histopathology is the gold standard for the diagnosis of FMT, allowing accurate characterization of the cell population and evaluation of histological features associated with its biological behavior [21]. The first International Histologic Classification of Mammary Tumors of Domestic Animals was published in 1974 to serve as the global standardization of tumor nomenclature in domestic animals. This classification was largely centered on descriptive morphology and less so on histogenesis [45]. In this classification, mammary lesions were divided into six groups: carcinoma, sarcoma, carcinosarcoma, benign tumors, unclassified tumors, and dysplasias. Further subdivisions of each group are displayed in Table 1.

The classification system was revised and updated in 1999 [46]. This second edition encompassed a less complex species-specific histological classification for FMT, based solely on the descriptive morphology, which accommodated four main groups of lesions: malignant tumors, benign tumors, unclassified tumors, and hyperplasias/dysplasias (Table 2) [46]. Even though a case of anaplastic FMC had already been described [47], this classification system still did not mention this tumor type. Benjamin (2001) noted that this classification offered mostly short descriptions lacking detail, which might add further subjectivity during the interpretation of the lesions [48].

Over the following 20 years, several new histological subtypes have been proposed, including inflammatory mammary carcinoma [49], lipid-rich carcinoma [50], invasive micropapillary carcinoma [51], and complex carcinoma [52]. When compared to other FMCs, invasive micropapillary carcinomas were associated with aggressive clinicopathological characteristics (i.e., higher nuclear grade, higher histological grade, muscle invasion, LVI, and nodal metastasis) [51].

In 2019, Zappulli et al. proposed a third edition of the classification, comprising more detailed descriptions, images, and information on differential diagnoses and biological behavior. Moreover, a standardization of the malignancy criteria was also proposed, indicating that the following characteristics should be considered when lymphatic invasion and regional lymph node metastasis are not detected: tissue architecture (i.e., loss of tubular pattern, nest formation, pluristratification with dysplasia and nuclear atypia), evident nuclear and cellular pleomorphism, mitotic count ≥ 6 per 2.37 mm^2^, randomly distributed intratumoral necrosis, and infiltrative behavior [21]. Further complexity was added to the histological classification of FMT, which were distributed over eight main classes: hyperplasias/dysplasias, benign epithelial tumors, malignant epithelial tumors, malignant special-type epithelial tumors, malignant mesenchymal tumors, carcinosarcoma, hyperplasias/dysplasias of the teat, and tumors of the teat (Table 3). For a few selected subtypes, a percentage threshold was indicated when a specific histological pattern was predominant, thereby reducing the subjectivity in classifying these cases. Accordingly, the recommended cutoff percentages are as follows: a tubulopapillary pattern is defined when >20% of the tubules exhibit intraluminal papillae, and both tubulopapillary and tubular carcinomas are diagnosed when >50% of the pattern predominates over a solid pattern. Adenosquamous carcinoma is considered when ≥25% of the pattern exhibits squamous differentiation, whereas lipid-rich carcinoma is considered when >50% of the tumor consists of vacuolated cells. Additionally, an anaplastic pattern should be reported even when present in a small proportion of the tumor, with hybrid reporting recommended (e.g., solid carcinoma with focal anaplastic carcinoma). Although no specific proportion is defined, the diagnoses of both comedo and anaplastic carcinomas are described as requiring the respective histological pattern to be predominant [21].

**Figure 1:**
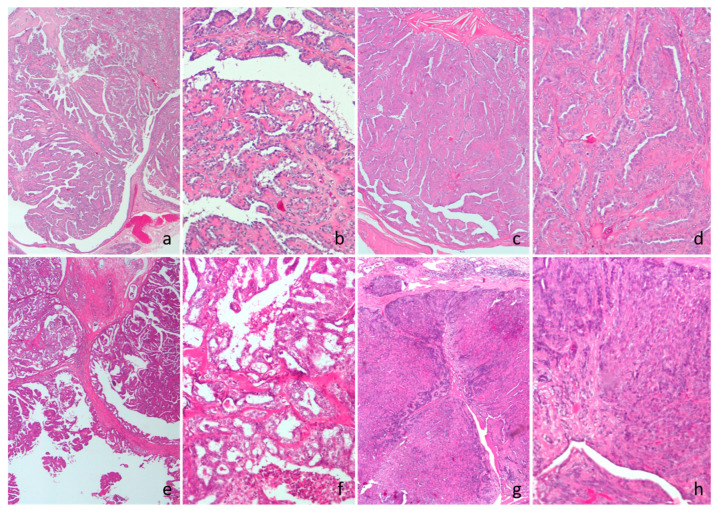
Cat, hematoxylin and eosin. (**a**,**b**) Intraductal papillary adenoma. (**c**,**d**) Ductal adenoma. (**e**,**f**) Intraductal papillary carcinoma. (**g**,**h**) Ductal carcinoma.

While the 1999 classification describes cribriform carcinomas as solid tumors with sieve-like openings [46], the current system excludes cribriform carcinomas as an individual entity and includes them under tubular carcinomas, identifying the characteristic apertures as small tubular lumina. The term carcinoma in situ is currently discouraged, and its substitution with epitheliosis or ductal/lobular hyperplasia with severe atypia is recommended [21]. Accordingly, a recent review on the terminology highlighted the lack of uniformity across different authors and the need for standardization [53]; some authors use the term in situ to convey the idea of a non-invasive tumor, irrespective of its histological subtype [54,55,56], whereas the 1999 WHO classification defined it as a diagnostic entity [46]. Moreover, even though they are included in the contents of the series, non-simple tumors were excluded from the FMT 2019 classification [21]; complex carcinomas reported in the literature and complex adenomas were classified as ductal-associated tumors (Figure 1). Additionally, formerly known fibroadenomas are now categorized as focal fibroadenomatous changes [21]. On the other hand, Sammarco and colleagues (2020) recently identified four FMT displaying a similar morphology and immunohistochemical labeling as canine mammary carcinoma and malignant myoepithelioma, demonstrating the occurrence of biphasic FMC [57]. Although the current histological classification of FMT includes prognostic remarks, several subtypes still lack follow-up information, warranting the need for research confirming its prognostic value.

A recent multicentric ring study aimed at evaluating the interobserver variability in the classification of canine mammary lesions noted that while there was general consensus in categorizing the biological behavior of the lesion (i.e., hyperplasia, benign tumor, or malignant tumor), agreement among the 15 participating pathologists regarding the specific histological subtype was deemed moderate [58]. While the first two editions of the FMT classification system were not overly complex, the recent update has contributed to a closer alignment with the more intricate canine classification. Hence, it is crucial to standardize the application of the classification system in these tumors, as demonstrated by Papparella and colleagues (2022), regardless of the species.

### 2.2. Histological Grading Systems

Histological grading of human breast cancer was first proposed in 1925 as a three-tier grading scheme entailing the assessment of eight morphological features [59] and served as a starting point for the development of contemporary grading schemes. However, despite currently providing important prognostic and predictive value, histological grading of breast cancer was not readily adopted due to its complexity, subjectivity, and limited treatment options at the time [60].

In 1957, the first numerical grading system was introduced [61], based on which, 34 years later, Elston and Ellis (EE) published what would become the gold standard for the histological grading of invasive human breast cancer [62]. The EE histological grading system has been widely applied by veterinary pathologists and researchers in the grading of FMC [20,27,30,55,63,64,65] and encompasses a semi-quantitative assessment of three morphological features, namely tubule formation, nuclear pleomorphism, and mitotic count (Table 4). A numerical score between 1 and 3 is allocated to each parameter, and the total score is used to attribute the tumor grade: grade I (3–5 points; well-differentiated), grade II (6–7 points; moderately differentiated), or grade III (8–9 points; poorly differentiated) [62]. According to literature reports, FMC grade II is usually the most prevalent, accounting for up to 58.3% of the cases [14,56,63,64,66]; however, one study reported grade III as the predominant histological grade, encompassing 59.2% of the cases [30]. While most solid carcinomas are moderately to poorly differentiated, tumors exhibiting tubulopapillary patterns are mainly well to moderately differentiated [27,63,64]. Furthermore, EE grade I carcinomas have predominantly been observed in smaller tumors (median 1.1 cm) and younger (median 10 years) cats, whereas grade III carcinomas were more frequent in larger tumors (median 3.0 cm) and older cats (median 12 years) [27].

In 2012, Matos and colleagues highlighted the potential bias of using adaptations from human histological grading systems in veterinary tumors, and thus emphasized the need for standardized species-specific grading methods [67]. Soon after that, Mills and co-workers (2015) sought to review and modify the EE method and developed a novel grading system for FMC in an attempt to improve the prognostic value of routine histological assessment of FMC. Hence, three histological grading methods were proposed, namely mitotic modified Elston and Ellis (MMEE), revised Elston and Ellis (REE), and Mills novel grading system (MGS) (Table 4).

The MMEE includes the same criteria as the classic EE method; however, mitotic count thresholds were modified to better accommodate the high mitotic activity observed in FMC, which often leads to the underrepresentation of grade I tumors. In the REE, besides modifying the mitotic count thresholds, assessment of nuclear pleomorphism was replaced by nuclear form scoring, and LVI was included, adding a potential extra point to the total score. For the nuclear form scoring, deviations from the normal round to oval smooth nuclear contour were considered abnormal nuclear forms. Finally, Mills and co-workers (2015) proposed a novel grading system encompassing the independent prognostic factors of their study: nuclear form, mitotic count, and LVI. Whereas in the EE, MMEE, and REE grading systems, a score between 1 and 3 is assigned to each parameter, with the only exception being the LVI feature in REE, in the MGS, the score is either 0 or 1 [23].

**Figure 2:**
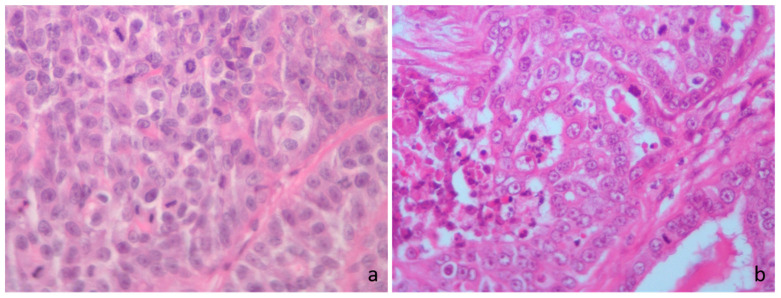
Cat hematoxylin and eosin. (**a**) High mitotic count score, irrespective of the mitotic thresholds; >71 mitoses in 2.37 mm^2^. (**b**) High mitotic count score according to the Elston and Ellis (EE) grading, low mitotic score according to Mills et al. [23] Mitotic-Modified EE (MMEE), Revised EE (REE) and the novel grading system (MGS), high mitotic score according to Dagher et al. [14] cutoff for MGS and low mitotic score according to Granados-Soler et al. [70] cutoff for MGS; 34 mitoses in 2.37 mm^2^.

Several authors have since used and evaluated the proposed FMC-adapted grading systems, mostly supporting their potential prognostic value [14,65,68,69]. However, while Mills and co-workers (2015) drew mitotic count cutoffs from the tertile boundaries (MMEE and REE) and median (MGS) of their study cohort [23], Dagher and colleagues (2019) derived their mitotic count cutoffs by adapting the MMEE and REE mitotic thresholds to their microscopic field diameter, and the MGS mitotic cutoff was used according to the receiver-operating characteristic (ROC) curve for the 2-year overall survival (OS) of their own cohort [14], resulting in very different thresholds used. The same approach was later taken by Granados-Soler et al. (2020), resulting in yet another mitotic count cutoff [70]. Moreover, the mitotic cutoffs used by Soares et al. (2016), Chen et al. (2020), and Rosen et al. (2020) to assign each histological grade are unclear [65,68,69]. Even though an adaptation of the mitotic thresholds was pointed out as a good strategy to improve prognostic value in FMC cases [14], a consensus of which thresholds to use in routine histological grading is warranted (Figure 2). Furthermore, given the current literature on interobserver reproducibility of grading canine mammary carcinomas, which uses the same morphological features as the EE system [58,71], perhaps the inclusion of morphological features that are less prone to bias and discordance should be considered for the grading frameworks. In this regard, the incorporation of LVI as observed in the proposed REE and MGS schemes could be a good strategy to improve the prognostic value of histological grading.

## 3. Prognostic Evaluation

### 3.1. Epidemiological and Clinical Parameters

#### 3.1.1. Age

Overall, age has not been proven to be a consistent prognostic factor in FMT. Early research determined a weak association between age and survival, with elderly queens having shorter survival regardless of the presence of distant metastasis [22]. Later on, another study found a relationship between old age and worse tumor-specific survival (TSS) and disease-free interval (DFI); however, significance was lost upon multivariate analysis. Additionally, a higher incidence of poorly differentiated tumors was observed in older queens [27]. In contrast, most published papers found no prognostic value related to age [8,10,12,14,23,30,40,63,64,72,73,74].

#### 3.1.2. Breed

Mixed-breed cats are often overrepresented in FMC literature [25,26,40,75,76]. Initial studies in North America observed a 2-fold increased risk of Siamese cats developing mammary carcinomas when compared to other breeds [19,33], with an apparently similar tendency being observed in Japan [10]; however, there may have been an overrepresentation of the breed in these studies [31]. In a small data set, Siamese cats (*n* = 3) with FMC had significantly worse prognosis, with a median DFI and OS of 190 and 298 days, compared to 324 and 474 days in Domestic Shorthairs, respectively [75]. Weijer and Hart (1983) observed the presence of smaller primary tumors in long-haired cats, which, interestingly, also had significantly worse survival and more frequent lymph node metastasis; however, significance was lost in the multivariate analysis [22]. Recently, breed was an independent prognostic factor for DFI, with Domestic Shorthair cats presenting a 2.8-time higher risk of disease recurrence than pure-breed cats; however, the latter may have been underrepresented [26]. Notwithstanding, multiple reports found that breed had no significant influence on the prognosis of cats with FMC, regardless of gender [8,10,12,40,74].

#### 3.1.3. Reproductive Status/Contraceptive Administration

In a case–control study, ovariohysterectomy performed before 6 months, between 6 and 12 months, and between 13 and 24 months reduced the risk of development of FMC by 91%, 86%, and 11%, respectively. The median age of neutering in the diseased group was 48 months, whereas in the control group, it was 8 months [34]. Furthermore, compared to cats with FMC that were spayed at some point in their lives, those that remained intact had significantly shorter survival [14]. In contrast, according to multiple authors, reproductive status (intact vs. spayed) did not exert a significant impact on the disease progression and survival of queens with FMC [12,22,23,26,30,40]. However, the timing of ovariohysterectomy was not considered in these studies, which might justify the lack of significant results. Nonetheless, the mean age reported for neutering was 5.8–6.0 years of age [13,22], and in a recent independent study, only 4% of the diseased cats were spayed before 2 years of age [14].

Alternatively, exogenous progestogens have been used to prevent estrus and control reproduction in cats; however, high doses of these drugs seem to be associated with adverse effects, including mammary changes [77,78]. Studies have reported the occurrence of mammary gland hypertrophy, viz., fibroadenomatous change, following the administration of progestogens and deslorelin implants [79,80,81,82,83]. In an early study, fibroadenomatous change was observed in 52.9% (9/17) of the cats treated with progestogens, contrasting with 11.3% (11/97) of the untreated cats that presented the same lesion. Meanwhile, mammary carcinoma was the most prevalent lesion in the untreated group, affecting 73.2% of the cats [35]. Nonetheless, cats with a contraceptive history might represent up to 57.7% of felines with mammary carcinoma [8,22,26,30]. A couple of studies on FMC reported no significant differences in the survival time and DFI of cats with a history of contraceptives versus untreated cats, irrespective of gender [8,26].

According to the literature, between 30.7% and 62.7% of the intact queens with FMC were multiparous [13,22,34], whereas cats with no mammary tumors appeared to be mostly (89.1%) nulliparous [34]. Despite that, no association has been found between parity and FMC development, progression, and survival [22,34].

#### 3.1.4. Clinical Staging

The clinical staging of feline malignant mammary tumors was first adapted from human oncology in 1980 by Owen and colleagues to stratify cases based on tumor extension and prognosis, serving as a tool to support clinical decision-making in the management and treatment of the disease [84]. Since then, a slightly modified version of the TNM system has been widely adopted by veterinary practitioners [28,31]. Most authors agree on the significant prognostic value of assessing the clinical stage in FMC [10,14,23,26,27,30]. In an early study, queens with stages I, II, III, and IV exhibited a median survival time of 29, 12.5, 9, and 1 months, respectively [10]. Similarly, in subsequent studies, cats with stages I, II, and III survived a median of 16.2–24.0, 11.8–15.0, and 6.0–10.0 months, respectively [14,23,27], with an average 2-year survival rate of 41.3% for stage I and 0% for stage III [27]. A similar trend was noted relating to the DFI, with cats at stages I, II, and III surviving a median of 12.0, 14.0, and 3.0 months free of disease, respectively [27]. Furthermore, stage III queens presented a recurrence risk 2.4 times higher than those with stage I disease [26]. A significant influence of the clinical stage was reported on the DFI; however, while it remained an independent prognostic factor in the works by Petrucci and colleagues (2020, 2021), Seixas and co-workers (2011) failed to obtain similar results [26,27,30]. Although research often excludes stage IV cases, a recent study in metastatic FMC reported a median survival time of 44 days in such cases, and a median time to disease progression of 23 days after stage IV diagnosis [25]. In univariate analysis, most studies observed a significant impact of the TNM stage on the OS and TSS of diseased cats [10,14,23,26,27,30]; however, significance was lost after adjusting for other covariates [26,27].

Recently, in an attempt to enhance TNM stage prognostic precision, a study proposed the subdivision of stage III into three sub-categories (i.e., III_A_, III_B_, and III_C_), according to tumor size, extension of the tumor to surrounding tissues, and nodal status. This modified staging system was significantly associated with both DFI and OS; queens in stages III_B_ and III_C_ presented poorer outcomes than those in stage III_A_ [29].

#### 3.1.5. Regional Lymph Node Metastasis

While axillary lymphadenectomy is not typically included in the resection of thoracic mammary glands, the inguinal lymph nodes are usually excised when resecting the caudal mammary glands [85]. Even so, feline cranial abdominal mammary glands can drain to the axillary lymph center [86,87] and, in turn, the caudal thoracic glands can drain to the inguinal lymph nodes [87]. This knowledge is particularly important during the clinical staging and planning of the surgical procedure, given that neoplastic cells are detected in 23–79% of the regional lymph nodes of animals diagnosed with FMC [4,14,22,24,26,27,28,29,30,40,65,88]. Nodal status on microscopic examination is one of the most dependable prognostic factors in FMC, as the majority of studies observed an inverse association between the presence of lymph node metastasis and survival time [14,22,23,27,29,30], retaining its significance when adjusted to other clinicopathological features [22,27]. Nodal status has also been significantly correlated with DFI [27,29,30]; however, it was not an independent prognostic factor. Interestingly, in the univariate analysis of the other two studies, lymph node metastasis predicted DFI, but not survival time [26,65]; however, both studies apparently included cats with surgery as the only treatment, as well as cats with surgical and adjuvant chemotherapy treatment, which might have influenced DFI and survival times. Accordingly, cats with lymph node involvement had a median survival time of 9 months, whereas those with negative nodal status survived a median of 16 months [23]. Similar results were obtained by Dagher and colleagues (2019), who reported a median survival time of 7.7 months in felines with lymph node metastasis and 13.3 months in felines with negative or unknown nodal status [14]. In one study by Seixas and co-workers (2011), cats with lymph node involvement displayed disease progression within 8 months after surgical treatment [27].

#### 3.1.6. Surgical Technique

Different surgical techniques, from lumpectomy to bilateral mastectomy, can be applied depending on several aspects such as tumor size, location, and patient status [85]. In 1983, Weijer and Hart called attention to the fact that a median of 5 months (ranging from 0 to 72 months) elapsed between detection of the tumor by the tutor and the first surgical procedure, contrasting with a less than one week interval between detection by the veterinarian and the first surgery, thus highlighting the importance of a timely veterinary appointment in order to act earlier in the disease [22]. Over the years, studies have reported a wide range of survival and DFI times, and taken together, the results suggest that cats with FMC treated with more conservative procedures have worse prognoses. The median survival time for cats treated with partial/regional, unilateral, and bilateral mastectomy has been reported to be 1406, 414–473, and 1140 days, respectively [4,28]. Similarly, the reported DFI ranges of 205–428, 115–474, and 510–917 days for cats that underwent partial, unilateral, and bilateral mastectomies, respectively [4,28,75,89]. In a cohort of 90 cats, those undergoing radical mastectomy had a significantly longer DFI than those treated with partial mastectomy [74]. In the same manner, disease progression and consequent death were significantly more likely to occur, and at an earlier time, in cats submitted to unilateral mastectomy than those treated with bilateral mastectomy [4]. On the other hand, unexpectedly, in the univariate analysis by Weijer and Hart (1983), cats treated with more conservative surgery had a better prognosis [22]. However, most cats treated with aggressive surgery had lymph node metastasis; also, significance was lost on multivariate analysis [22]. Nevertheless, several studies failed to find significant differences in the survival of cats undergoing different surgical approaches [8,10,26,65]. Identical non-significant results were obtained in a small cohort after comparing the DFI and OS of both partial versus radical mastectomy and unilateral versus bilateral mastectomy [75].

In a related context, the type of surgery might have an influence on the histological completeness of the margins, as one would assume that a more radical approach has a higher chance of completely excising the tumor. Thus, margins might be an important variable to consider when assessing the prognostic value of the surgical procedure in future studies. Nonetheless, a few authors found a significant association between surgical margins and clinical outcome when comparing complete versus incomplete and complete versus narrow/incomplete surgical margins [22,26,65]. However, no significant differences regarding the outcome of cats with FMC were observed when considering three margin categories (i.e., complete, narrow, and incomplete) [26].

#### 3.1.7. Tumor Size

Tumor size has been widely studied as a prognostic factor in FMC, and although several studies report a significant influence on the animals’ survival, the results are not consistent among different authors [8,10,14,23,26,27,40,64,65]. Early research in FMC has measured tumor volume, which showed a significant association with survival [13] and DFI [74]. Cats with smaller tumors (0–8 cm^3^) had a 1-year survival rate of 50%, whereas those with larger tumors (>65 cm^3^) had a 1-year survival rate of 8% [13]. Curiously, Weijer and Hart (1983) assessed both tumor volume and diameter, and despite obtaining a significant association between both variables and survival outcome in the univariate analysis, upon multivariate analysis, significance was lost for tumor volume, while tumor diameter (grouped as 1, 2, 3–5 or ≥6 cm) arose as an independent prognostic factor [22]. Since then, researchers have adopted tumor diameter as the standard tumor size indicator, which is used for TNM staging. Ito and colleagues (1996) used a cutoff of 3 cm, noting that cats with tumors ≤3 cm survived a median of 9 months, whereas those with larger tumors survived a median of 5 months [10]. In contrast, the same cutoff did not yield significant results in a subsequent study, even though cats with smaller neoplasms had a median survival of 21 months, compared to a median of 12 months in cats with larger neoplasms. The same study also observed a lack of significant results when categorizing tumor diameter as <2, 2–3, and >3 cm [12]. Conversely, other authors found a significant link between the same three-tier system and prognosis, observing worse outcomes in cats with larger tumors [8,29]. Likewise, queens with a tumor diameter of <2 cm survived significantly longer, with a median TSS of 18 months and an average 2-year survival rate of 34%, while those with tumors measuring 2–3 cm and >3 cm survived a median 8 and 5 months and had a 2-year survival rate of 25% and 0%, respectively. Similar results were obtained regarding the DFI. Even so, tumor size was not an independent predictor of TSS and DFI in the multivariate analysis [27], contrasting with the aforementioned results by Weijer and Hart (1983) [22]. Several other authors did not find any significant results regarding tumor diameter, both as a categorical variable [23,26,64] and as a continuous variable [65]. Although it is reasonable to assume that tumor size was measured at some point before fixation in these studies, this is not explicitly stated in most of the methods.

Tumor size measured on the hematoxylin and eosin (HE)-stained slides, considering a cutoff of 2 cm, was also a negative prognostic predictor [14,56]; cats with smaller tumors survived a median of 12.4 months, compared to 7.7 months for cats with larger tumors [14].

Inconsistencies between studies may be attributed to the methods employed in each study, such as the measurement timing (i.e., before excision, after excision and before formaldehyde fixation, after formaldehyde fixation, or on histological slides), size indicator (i.e., volume or diameter) and considered thresholds. Collectively, these findings highlight the need to standardize tumor measuring methods, as previously pointed out by other authors [15].

#### 3.1.8. Tumor Ulceration

Ulceration can be a common finding in FMC, being present in 7.5% to 45% of the cases [22,24,88,90]. In an early study, cutaneous ulceration was inversely associated with survival; however, significance was lost upon multivariate analysis, probably because ulceration was strongly associated with the presence of necrosis [22]. In recent studies, cutaneous ulceration was independently associated with OS [29,56]. Furthermore, ulceration also seemed to affect DFI [29] and TSS on univariate analysis, though its significance for TSS was lost after adjusting for other clinicopathological features [56]. Other authors found no significant prognostic value of cutaneous ulceration in FMCs [26,30,40].

#### 3.1.9. Others

The prognostic value of other FMC clinical variables such as the number of lesions, location, and adherence to the underlying tissue has been considered by different studies; however, none of the features held relevant results [13,22,26,30,64].

### 3.2. Pathological Parameters

#### 3.2.1. Tumor Growth

While benign tumors are typically well-demarcated, presenting expansive growth, malignant neoplasms often lack clear boundaries, displaying infiltrative growth [21,91,92]. Accordingly, malignantly transformed cells frequently do not abide by normal anatomical barriers, breaching the basement membrane and progressively invading surrounding tissues [91,92]. Infiltrative tumor growth is commonly found in FMC, being reported in up to 91% of the tumors [22,56,76]. In one study, a significant relationship between infiltrative growth and survival was found; however, significance was lost after being adjusted for other covariates. No association was found between an infiltrative growth and DFI [22]. Another study reported a 2-year survival rate in all the cats diagnosed with non-infiltrative in situ carcinomas (*n* = 3) [76]. In a similar manner, Chocteau and colleagues found that cats with in situ carcinomas had significantly longer DFI, OS, and TSS than those presenting invasive carcinomas [56].

#### 3.2.2. Histological Subtype

The influence of the histological subtype on the survival of animals with FMC has been investigated by several researchers over the years. While early studies [55,63,72,73] did not yield significant results, it is important to consider that these authors classified FMT according to the first histological classification of mammary gland lesions of domestic animals, which lacked a feline-specific classification [45]. Following the updated species-specific histological classification [46], in a cohort encompassing tubulopapillary, solid, cribriform, and squamous cell carcinomas, cats diagnosed with tubulopapillary carcinomas survived significantly longer compared to those with solid and cribriform subtypes, with respective median TSS times of 21, 10, and 8 months [23]. Dagher and co-workers (2019) reported a median survival time of 12 and 8.9 months for cats with cribriform and solid carcinomas, respectively, with the latter group including adenosquamous, squamous cell, and anaplastic carcinomas [14]. Univariate analysis showed that cats in the solid carcinoma group had a significantly poorer OS prognosis; however, the result was not significant when pertaining to the TSS. Moreover, no significant differences were reported between mucinous or tubulopapillary carcinomas and cribriform carcinomas [14]. Another study found no influence in DFI or OS when considering four histological groups (i.e., tubulopapillary, cribriform, solid, and other types of carcinomas) [26]. Over time, new histological subtypes with potential prognostic significance have been described and were recently included in the third edition of the international classification for FMT [21]. Queens with invasive micropapillary carcinomas had significantly poorer clinical outcomes, presenting a median TSS and DFI of 4 and 5 months, respectively, versus 10 months (both TSS and DFI) in cases diagnosed with other types of FMC [51]. On the other hand, cats with complex carcinomas—which can reasonably be assumed to fall within the ductal-associated category [21,93]—survived significantly longer, presenting a mean OS of 32.6 and a 2-year survival rate of 80%, compared to a mean OS of 15.5 months and a 2-year survival rate of 21% in cases with other FMC [52,94]. Lastly, in a study considering two distinct groups—solid carcinomas (solid and comedocarcinomas) and tubulopapillary carcinomas (tubulopapillary and intraductal papillary carcinomas)—queens with solid carcinomas had significantly shorter TSS and DFI [70]. Notwithstanding, to the author’s best knowledge, the implemented changes in the histological classification of FMT have yet to be properly analyzed in a cohort diagnosed accordingly.

#### 3.2.3. Histological Grading

The EE grading system has been extensively used in FMC, with numerous studies supporting its prognostic value in this species [26,27,30,55,56,63,64,68,70]. In an early study exploring the suitability of the EE grading system in FMC, the 1-year post-surgical survival rate was 100%, 42.4%, and 0% for FMC grades I, II, and III, respectively, demonstrating a good predictive value, particularly in the case of grade I and grade III tumors [55]. Correspondingly, in a cohort with a post-surgical follow-up of at least 2 years, 37.5%, 52.2%, and 100% of the cats with FMC EE grades I, II, and III, respectively, died of tumor-related causes [63]. The same authors obtained similar results in a subsequent study, with the biggest difference being noted in the EE grade II group, which had a 70.3% tumor-death rate at the end of the 2-year follow-up period [64]. Cats with EE grade III FMC typically had significantly shorter OS [27,29,63] and an increased risk of death of up to 3.1 times higher, when compared to those with grade I tumors [26]. Indeed, queens with grade III carcinomas had a median survival of 6 months, when compared to a median survival of 36 months for those with grade I tumors. Histological grade has emerged as an independent prognostic predictor for DFI, OS, and TSS [26,27,29,56]. Nonetheless, the results are not always consensual among authors; in one of the aforementioned studies, histological grade was significantly associated with DFI and TSS in the univariate analysis, but not in the multivariate analysis [56]. Similar results were observed by an independent study regarding the prognostic value of grade III for DFI and TSS [30]. Dagher et al. (2019) and Mills et al. (2015) have observed a significant difference between the survival of cases with EE grade III and grade II, despite the lack of an overall association between the histological grade and survival in the latter [14,23]. When applied to the same cohort, the MMEE grading system exhibited a significant association with TSS, with the median survival of FMC MMEE grades I, II, and III being 27, 14, and 13 months, respectively. However, upon separate analysis of the grades, no significant differences were found between grades II and III [23]. By using an adapted mitotic count threshold, two other studies also obtained a statistically significant association between histological grade and OS, TSS, and DFI [14,70]. Upon multivariate analysis, cats with MMEE grade II and III displayed a significantly worse OS than those with grade I, independently of tumor size and positive nodal status, and a worse TSS after adjusting for positive nodal status [14]. The REE histological grading has proven to be significantly prognostic [14,23,68], with higher histological grade FMC being inversely associated with OS and TSS, independently of tumor size and presence of lymph node metastasis [14]. Indeed, cats with REE grades I, II, and III presented a median survival of 29, 12, and 5 months, respectively, though upon statistical analysis, no significant differences were found between grades II and III [23]. Curiously, when assessing the REE grading excluding the LVI parameter, a significant difference between the OS of grades I and II was found, as opposed to a non-significant difference between grades I and III. Additionally, the REE grade did not affect TSS in this analysis [14].

Regarding the MGS grading, studies have reported a significant association with both survival and DFI [23,65]. Cats with MGS grades I, II, and III had a median survival of 31, 14, and 8 months, and an 18-month survival rate of 82%, 37%, and 18%, respectively [23]. Similarly, Rosen and colleagues (2020) reported a median of 198.5 days (approximately 6 months) in cats with MGS grade III versus a median of 995.5 days (approximately 33 months) in a group including both MGS grades I and II [65]. Dagher and co-workers (2019) aimed to validate the MGS grading using a mitotic count cutoff adapted to the corresponding study’s cohort and observed a significant difference between OS and TSS of cats with MGS grade I and those with grade III, but the same was not true when comparing MGS grades I and II. After merging MGS grades II and III, the group had a significantly poorer OS and TSS when compared to MGS grade I, regardless of tumor size and positive nodal status [14]. In contrast, another study apparently obtained non-significant results using the MGS grading method [68].

All the histological grading systems discussed above seem to hold potentially important prognostic values; however, inconsistencies during the assessment of the different parameters may have led to somewhat conflicting results. A study on canine mammary tumors (CMT) evaluating the interobserver agreement in assigning EE grading scores found the highest agreement in the evaluation of tubule formation, followed by mitotic count and nuclear pleomorphism [71]. Indeed, the subjective nature of scoring nuclear pleomorphism and the variability in selecting areas for mitotic counting likely contribute to interobserver variability when grading mammary tumors [15,71]. Moreover, although the REE and MGS gradings aim to reduce subjectivity by replacing the nuclear pleomorphism feature with nuclear form, the latter remains subjective and would benefit from a clearer definition of the methodology.

It becomes evident that there is still an ongoing lack of consensus regarding the most appropriate histological grading framework for FMC, as previously noted [95]. Given the differing strengths of each grading framework and the uncertainty surrounding the mitotic count thresholds, the authors recommend the inclusion of both EE and MGS grades in the diagnostic report, in line with earlier suggestions [95]. This approach will allow veterinary clinicians to interpret tumor characteristics through different lenses. While the EE grading scheme had been validated by different studies, the MGS framework, in particular, incorporates LVI—an important marker of tumor invasion—and a seemingly less subjective parameter—nuclear form—offering a potentially improved prognostic accuracy. An alternative strategy to enhance EE prognostic accuracy could be the adaptation of the veterinary Nottingham Prognostic Index (vet-NPI) [96,97,98]—which combines tumor size, lymph node or LVI status, and histological grading—to FMC, and its inclusion in the diagnostic report as additional data.

#### 3.2.4. Tubule Formation, Nuclear Pleomorphism, Nuclear Form, and Mitotic Count

As histological grading systems incorporate several morphological parameters, their individual prognostic value has also been under the scope of several researchers. The OS and TSS of cats with different scores of tubule formation do not seem to significantly differ [14,23]; only a weak association has been reported between the TSS of cats with <10% and >75% tubule formation [23].

Nuclear pleomorphism was inversely associated with survival [13,14,22,23]. Cats with FMC that had marked nuclear pleomorphism survived a median of 5.4 months, while those that had a nuclear pleomorphism score of 1 or 2 lived a median of 18.5 and 14.3 months, respectively [14].

When evaluating the nuclear form, cats with ≤5% of abnormal nuclei survived a median of 21 months, compared to a median of 12 months in those with an abnormal score of >5%. Overall, the difference in TSS times was significant; however, on separate analysis (≤5% vs. 5–25%, 5–25% vs. >25%, and ≤5% vs. >25%), a statistically significant difference was obtained between the ≤5% and 5–25% groups, but not between the remaining pairs [23]. No differences were found between abnormal nuclear form categories and clinical outcomes in a cohort of 342 cats [14].

Mitotic count on FMC has been considered a significant prognostic value in univariate and multivariate analyses [22,23]. The 1-year survival rate in cats with FMCs exhibiting lower mitotic counts was significantly higher than those with higher numbers of mitotic figures [13]. While cats with a mitotic count > 62 in 10 consecutive high-power fields in the most mitotically active area survived a median of 9 months, those with a mitotic count ≤ 62 survived twice as long [23]. The number of mitotic figures as a continuous variable also demonstrated a significant predictive value for both OS and DFI [65]. On the contrary, in an independent study considering a cutoff of 33 mitotic figures (≤33 or >33), no significant associations were found regarding both the OS and TSS [14]. A consensus on the optimal grading scheme and mitotic count thresholds used in routine diagnostic, as well as a standardization of the assessment of each grading parameter, is essential, as it greatly impacts the resulting histological grades and potential prognosis. In this sense, and due to variations in microscope field areas, recent efforts have aimed towards the standardization of the mitotic count reporting within a total area of 2.37 mm^2^—an approach that future research will follow in order to facilitate cross-study comparisons [99,100].

In a related context, cats with FMC displaying a mitotic index above 72% had significantly poorer survival than those with a lower proliferative activity [76,101], with the variable remaining an independent prognostic factor on multivariate analysis [76].

#### 3.2.5. Histological Staging

In 2002, Preziosi and collaborators devised a histological staging system for FMC, based on previous works on CMT, encompassing three tiers according to tumor invasiveness: stage 0 (non-infiltrating carcinoma/carcinoma in situ), stage I (carcinoma with stromal invasion), and stage II (carcinoma with LVI and/or lymph node metastasis). Upon survival analysis, cats with stage I carcinomas survived significantly longer than those with stage II carcinomas, with the variable remaining significant in the multivariate analysis [76]. Expanding upon this, in 2019, Chocteau and colleagues proposed another histological staging system for FMC including five tiers: stage 0 (carcinoma In Situ, confirmed by p63+ immunohistochemistry), stage I (invasive, tumor size ≤ 2 cm, with negative/unknown nodal status and absent LVI), stage II (invasive, tumor size > 2 cm, with negative/unknown nodal status and absent LVI), stage IIIA (invasive, tumor size ≤ 2 cm, with positive nodal status and/or LVI), and stage IIIB (invasive, tumor size > 2 cm, with positive nodal status and/or LVI). Survival analyses revealed a significant association with DFI, OS, and TSS, even though the Kaplan–Meier survival curves for stages II and IIIA were poorly separated. Furthermore, the histological staging remained a predictive factor for OS and TSS after adjustment to other clinicopathological features [56]. Notwithstanding these results, subsequent research on histological staging has been limited.

#### 3.2.6. Lymphovascular Invasion

Lymphovascular invasion is considered one of the most reliable prognostic indicators for FMC [15], and has been reported in 22.9% to 69.6% of the cases [13,14,23,26,27,30,68,88,102]. Queens with LVI presented a median survival time of 7–8 months, compared to a median of 16–36 months in those without lymphovascular emboli [14,23,27]; the average 2-year survival for queens with and without LVI was 6.7% and 59.3%, respectively [27]. Similarly, in a cohort of 27 male cats with FMC, those exhibiting lymphatic invasion survived a median of 195 days (approximately 6.5 months), contrasting with the median survival of 863 days (approximately 28.8 months) in animals without lymphatic permeation, uncovering a significant difference between the two groups [8]. Overall, the presence of lymphovascular emboli has been a consistently significant outcome predictor [14,23,26,27]. Nevertheless, when evaluating the association between LVI and TSS, Dagher and co-workers (2019) obtained statistically significant results [14], whereas Petrucci and colleagues (2021) did not [30]. Moreover, in two independent studies considering a 1-year survival cutoff, no differences in the survival of cats with and without LVI were observed [40,68]. On the other hand, Cox proportional hazard analysis revealed LVI to be an independent outcome predictor of survival [23,26] and DFI [27]; in these studies, queens with LVI presented a hazard ratio of 1.7 to 4.5 compared to those without invasion [23,26,27]. Even though other studies observed a significant association between LVI and DFI in the univariate analysis [26,30], significance was lost in multivariate analysis [26].

In one study regarding individual lymphatic and blood vascular invasion assessment, lymphatic permeation was present in 22.9% of the cats with FMC, contrasting with 8.2% that had blood vessels with neoplastic emboli [13]. These results suggest that FMCs are more likely to spread via the lymphatic system rather than the vascular system. Curiously, in human breast cancer, blood vascular invasion was negatively associated with both DFI and OS, remaining an independent predictor after multivariate analysis, as opposed to lymphatic invasion, which did not reveal a significant relationship with OS and lost its initial prognostic significance regarding the DFI in the multivariate analysis [103]. It is interesting to note that, in FMC, Rosen and colleagues (2020) observed that 20% (*n* = 3) of the cats with lymphatic invasion had a mean survival of 22 months post-surgery. Despite this, cats with lymphatic neoplastic emboli survived significantly less time than those without lymphatic invasion [13,22,65]. In one of these studies, however, significance was lost upon multivariate analysis, probably due to the fact that the presence of lymphatic emboli was strongly associated with regional lymph node metastasis [22]. In a related context, Preziosi and co-workers (2002) observed a significantly shorter survival in cases of FMC with neoplastic emboli and/or positive nodal status; the mean TSS in the group with invasion was 13.4 months, whereas in the group without invasion, it was 21.8 months [76]. Nevertheless, a separate evaluation of cats with emboli and lymph node metastasis was not performed, thus making it difficult to grasp their individual effect on the survival of the animals.

According to recently published veterinary cancer guidelines, it is important to distinguish between lymphovascular and pseudo-vascular invasion; however, it can be difficult to make the distinction, and the subject has yet to be evaluated in veterinary studies [104]. Pseudo-vascular invasion can occur during tumor manipulation, causing artifactual dislodgement of neoplastic cells into vascular spaces and mimicking the appearance of vascular invasion, even though the presence of these cells within vessels is not due to true tumor invasion [104,105,106]. In order to address this matter, the Veterinary Cancer Guidelines and Protocols group, drawing from human literature, recommends reporting whether LVI has been considered upon observation of strict criteria (i.e., thrombi adherent to intravascular neoplastic cells and/or tumor cells invading through a vessel wall and respective endothelium) or soft criteria (i.e., neoplastic cells within an endothelium-lined lymphovascular vessel with or without confirmation by immunohistochemistry) [104,106]. Furthermore, it is advocated that future veterinary studies provide detailed data regarding lymphatic versus blood vessel invasion and intratumoral versus peritumoral invasion [104], though the College of American Pathologists’ Protocol for the examination of human invasive breast carcinoma considers the former unnecessary [107]. Indeed, data regarding LVI in FMC literature can be uncertain about which vascular structures (i.e., lymphatic versus blood vessels versus lymphovascular) were considered in the evaluation [40,76,102] and whether LVI was detected in intra- or peritumoral area [8,13,14,22,23,26,27,30,40,65,68,76]; therefore, an effort towards clarification and standardization when reporting such data is warranted.

#### 3.2.7. Molecular Phenotyping

Five different molecular subtypes of breast cancer have been recognized based on the expression patterns of a set of genes, entailing the following classifications: luminal A, luminal B, human epidermal growth factor (HER2)-positive, and basal and normal breast-like [108]. As a widely applicable and cheaper alternative, a surrogate approach based on immunohistochemical tests was later defined by the St Gallen International Expert Consensus Panel, based on four markers: estrogen receptor (ER), progesterone receptor (PR), HER2 protein expression, and Ki-67 index [109,110]. This molecular phenotyping of breast cancer has played a pivotal role in advancing breast cancer care by providing a more precise approach to diagnosis and serving as a foundation for current therapeutic strategies [111].

The same molecular subtypes have subsequently been described in FMC [112,113]. In FMC, the considered Ki-67 threshold can vary between studies [113,114]; however, one study seeking to determine the ideal Ki-67 cutoff through survival analysis identified 14% as the optimal value [115].

The luminal A subtype is characterized by ER and/or PR positivity, absence of HER2 expression, and low Ki-67 index. Luminal B subtypes are defined as positive for ER and/or PR, with luminal B/HER2-positive presenting positivity for HER2 and any Ki-67 index, while luminal B/HER2-negative exhibits absence of HER2 and high Ki-67 index [110,113]. Luminal B subtypes, specifically luminal B/HER2-negative, have been reported as the most frequent subtype in FMC [69,112,113]. While luminal A FMC have been associated with less aggressive morphological features, such as smaller tumor size and low-grade tumor malignancy, the luminal B/HER2-positive subtype has been related to high-grade tumors [113]. Furthermore, cats with luminal A carcinomas present the longest survival and DFI times when compared to other subtypes [69,113]. In one study, cats with luminal A carcinomas had a median TSS of 1308, contrasting with a median of 479 and 392 days in those with luminal B/HER2-positive and luminal B/HER2-negative, respectively. Furthermore, cats with luminal A carcinomas presented a median DFI of 421 days, and those with luminal B/HER2-negative had a median DFI of 315 days [69]. Similar findings were observed in a different small cohort, in which progressively shorter TSS and DFI times were reported in luminal A, luminal B/HER2-positive, and luminal B/HER2-negative carcinomas [70].

The HER2-positive subtype is characterized by HER2 positivity, absence of ER and PR, and any Ki-67 index. The triple-negative subtypes are typically negative for the expression of ER, PR, and HER2 with any Ki-67 index [110,113]. Triple-negative basal-like (TNBL) present positivity to cytokeratin (CK) 5/6 and 14, while triple-negative normal-like (TNNL) do not [112,113]. TNBL FMC has been related to aggressive clinicopathological features, such as larger and poorly differentiated tumors [113]. Moreover, cats with HER2-positive and TNBL carcinomas usually present shorter survival and DFI times when compared to other subtypes, with the latter commonly displaying worse outcomes [69,113]. Accordingly, the median TSS of queens with TNBL and HER2-positive carcinomas was 59 and 281 days, respectively. Similarly, the median DFI of cats with TNBL and HER2-positive carcinomas was 28 and 209 days, respectively [69]. In line with these findings, a recent study encompassing a small cohort reported progressively longer DFI and TSS in queens with TNBL, TNNL, and HER2-positive carcinomas [70].

#### 3.2.8. Proteomics

In recent years, interest in the oncological proteomics field has increased, aiming to enhance disease characterization and monitoring, support diagnostic and prognostic assessments, and identify new therapeutic targets [116]. The first steps in FMC-related proteomics have already been taken, with a few studies already having researched serum and tissue peptidomes. A preliminary case–control study on non-metastatic FMC observed an association between several serum proteins—particularly, apolipoprotein A-II and apolipoprotein B—and the occurrence of the disease, suggesting their potential as early biomarkers for FMC [117]. A subsequent study on serum peptidomes identified proteins specific to non-metastatic FMC and metastatic FMC. While in the non-metastatic group, these were proteins that have been linked to antagonistic pro- and anti-proliferative functions (i.e., staufen homolog 2 (STAU2), bromodomain adjacent to zinc finger domain 2A (BAZ2A), gamma-aminobutyric acid type A receptor subunit epsilon (GABRE), complement C1q like 2 (C1QL3) and erythrocyte membrane protein band 4.1 (EPB41)), the metastatic-exclusive ones were proteins that have been predominantly linked to unfavorable prognosis (i.e., B-cell lymphoma 6 transcription repressor (BCL6), thioredoxin reductase 3 (TXNRD3), ceruloplasmin (CP), POU class 5 homeobox 1 (POU5F1), and laminin subunit alpha 1 (LAMA1)). Additionally, BAZ2A and GABRE were associated with chemotherapy, suggesting a potential role in therapy response [118]. The same group also performed an analysis on FMC tissues and found an upregulation of several proteins (i.e., interleukin 18 receptor 1 (IL18R1), rRNA adenine N(6)-methyltransferase (DIMT1), odontogenesis-associated phosphoprotein (ODAPH), immunoglobulin heavy chain variable (IGHV), gasdermin C (GDSMC), and phenylalanyl-tRNA synthetase (FARS2)) in metastatic cases, compared to both non-metastatic and healthy control groups [119]. Although available data remain limited, collectively, these findings highlight the potential of proteomics, particularly serum peptidomes, as a non-invasive tool for distinguishing disease stages and supporting the diagnosis, prognosis, and monitoring of FMC.

## 4. Conclusions

Reading through research on the clinicopathological features of FMT and their prognostic and predictive value often reveals a landscape of heterogeneity and lack of consensus among different authors. Notably, the lack of uniformity between methods assessing the same morphological features frequently leads to contradictory results. Future research must focus on the development of standardized and clearly defined evaluation methods, and, in this regard, the efforts of the Veterinary Cancer Guidelines and Protocols group are commendable and should serve as a starting point. Additionally, some studies appear to group together patients undergoing surgical treatment alone with those undergoing both surgery and adjuvant chemotherapy, which can complicate comparisons across studies and limit the reliability of the conclusions drawn regarding prognostic markers. That said, conducting veterinary studies, particularly oncological studies, presents several challenges that further complicate the establishment of robust conclusions. The fact that many animals end up being euthanized before reaching their natural endpoint is an important limitation, as it can skew survival data. Furthermore, follow-up depends on tutor compliance, often making it inconsistent and often times resulting in the loss of follow-up cases; sample size is frequently small, and most studies have a retrospective nature, limiting data acquisition and analysis. Consequently, these challenges may also act as limitations and influence the interpretation of several data points discussed in this review, especially given the heterogeneity across studies and the limited number of publications supporting some prognostic factors. Notwithstanding, several features have consistently emerged as prognostic factors in the available literature, namely clinical stage, regional lymph node status, lymphovascular status, EE histological grade, and tumor size. Given the lack of detail in how tumor size has been measured in the published literature, we recommend that the largest tumor axis be systematically measured using calipers before excision and post-fixation, during gross examination. Comparing pre- and post-fixation measurements, along with their respective prognostic value, could provide useful insights and help standardize this parameter in future studies.

As new editions of the FMT histological classification system are published, its prognostic value remains debatable, as only a few histological subtypes appear to be related to the outcome: does it genuinely aid in clinical decision-making, or is it a tool with limited practical value, with the sole purpose of providing a structure for the pathologists? Additionally, despite the important progress toward standardization in the latest histological classification of FMT, there is still room for improvement. One potential modification would be to consistently include and clarify pattern percentage cutoffs for all tumor subtypes, rather than limiting them to only a select few, as is currently the case—leaving the classification of other subtypes to interpretation. On the other hand, and perhaps more importantly, applying this approach without standardized rules to resolve classification challenges in tumors exhibiting multiple patterns that exceed cutoff thresholds, or in highly heterogeneous tumors that do not meet a specific threshold, risks introducing more diagnostic ambiguity rather than reducing it. Thus, it is crucial to establish clear criteria for resolving such overlaps, which may involve defining rules for subtype dominance, through approaches such as hierarchical reporting or hybrid classification in cases displaying significant heterogeneity.

Although aimed at accommodating the higher mitotic activity observed in FMC and diminishing the subjectivity of the EE grading system parameters (i.e., tubule formation and nuclear pleomorphism), the most recently proposed histological grading systems for these tumors still comprise morphological features prone to subjectivity. Additionally, the use of distinct cohort-adapted mitotic count cutoffs for the MGS grading across different studies raises concerns about its applicability to different cat populations, and especially in the diagnostic routine setting. Regardless, we believe the MGS grading scheme has the potential to attain strong prognostic accuracy, once it is validated using a commonly agreed-upon mitotic count threshold. Based on current literature, this could involve testing both the original cutoff of 62 mitoses and a lower cutoff around 34 mitoses in independent cohorts. Considering the importance of the histological grading in both research and clinical practice, it is surprising that the prognostic value of each individual parameter used to assign the histological grade has been scarcely investigated in FMC. To improve the clinical utility of grading frameworks, it seems essential to determine which specific parameters truly impact the survival of affected cats, rather than assuming that the composite grade fully reflects their prognosis.

In the global era we currently live in, perhaps a collaborative, multicentric, and multinational approach should be considered to allow the creation of a large sample size, ensuring a greater representation of uncommon histological subtypes and grades. This could provide more robust insights, fueling the discussion of whether these classifications and gradings accurately reflect tumor behavior and aggressiveness, and ultimately contributing to the refinement of these histopathological features.

Although the primary focus of this review is to summarize clinicopathological prognostic factors in FMT, it is important to acknowledge that some of these factors may have therapeutic implications. Prognostic indicators such as clinical stage, tumor size, histological grade, nodal and lymphovascular status, and molecular phenotyping can inform clinical decisions. Molecular phenotyping, in particular, is routinely used in human breast cancer to guide adjuvant therapy, but in FMC it remains underexplored and is not routinely performed. Further research and implementation of molecular phenotyping in routine FMC diagnosis have the potential to improve diagnostic precision and to guide and enhance the beneficial effect of adjuvant therapy strategies in this species.

## Figures and Tables

**Table 1:** Histological classification of feline and canine mammary gland lesions according to Hampe & Misdorp (1974) [45].

1974 Histological Classification	
I. Carcinoma A. Adenocarcinoma 1. Tubular (a) Simple type (b) Complex type * 2. Papillary (a) Simple type (b) Complex type 3. Papillary cystic (a) Simple type (b) Complex type * B. Solid carcinoma (a) Simple type (b) Complex type * C. Spindle cell carcinoma * (a) Simple type (b) Complex type D. Anaplastic carcinoma * E. Squamous cell carcinoma * F. Mucinous carcinoma II. Sarcoma A. Osteosarcoma B. Fibrosarcoma C. Combined sarcoma D. Other sarcomas III. Carcinosarcoma	IV. Benign tumors A. Adenoma B. Papilloma 1. Duct papilloma 2. Duct papillomatosis C. Fibroadenoma 1. Pericanalicular 2. Intracanalicular (a) Noncellular type (b) Cellular type 3. Benign mixed tumor * 4. Total fibroadenomatous change D. Benign soft-tissue tumor V. Unclassified tumors VI. Benign dysplasias A. Cyst 1. Nonpapillary 2. Papillary B. Adenosis C. Regular typical epithelial proliferation in ducts of lobules D. Duct ectasia E. Fibrosclerosis F. Gynecomastia G. Other non-neoplastic proliferative lesions 1. Noninflammatory lobular hyperplasia 2. Inflammatory lobular hyperplasia

* These tumor types were not observed in felines at the time.

**Table 2:** Histological classification of feline mammary tumors according to Misdorp et al. (1999) [46].

1999 Histological Classification	
1. Malignant tumors 1.1 Noninfiltrating (in situ) carcinoma 1.2 Tubulopapillary carcinoma 1.2.1 Tubular carcinoma 1.2.2 Papillary carcinoma 1.3 Solid carcinoma 1.4 Cribriform carcinoma 1.5 Squamous cell carcinoma 1.5.1 Adenosquamous cell carcinoma 1.6 Mucinous carcinoma 1.7 Carcinosarcoma 1.8 Carcinoma or sarcoma in benign tumor 2. Benign tumors 2.1 Adenoma 2.1.1 Simple adenoma 2.1.2 Complex adenoma 2.2 Fibroadenoma 2.2.1 Low-cellularity fibroadenoma 2.2.2 High-cellularity fibroadenoma 2.3 Benign mixed tumor 2.4 Duct papilloma	3. Unclassified tumors 4. Hyperplasias/Dysplasias 4.1 Ductal hyperplasia 4.2 Lobular hyperplasia 4.2.1 Epithelial hyperplasia 4.2.2 Adenosis 4.2.3 Fibroadenomatous change 4.3 Cysts 4.4 Duct ectasia 4.5 Focal fibrosis

**Table 3:** Histological classification of feline mammary tumors according to Zappulli et al. (2019) [21].

2019 Histological Classification	
1. Hyperplasia/Dysplasia 1.1. Duct ectasia 1.2. Lobular hyperplasia 1.2.1. Regular lobular hyperplasia 1.2.2. Lobular hyperplasia with secretory activity 1.2.3. Lobular hyperplasia with fibrosis 1.2.4. Lobular hyperplasia with atypia 1.3. Epitheliosis 1.4. Papillomatosis 1.5. Fibroadenomatous change 2. Benign epithelial neoplasms 2.1. Simple benign tumors 2.1.1. Simple adenoma 2.2. Non-simple benign tumors * 2.3. Ductal-associated benign tumors 2.3.1. Ductal adenoma 2.3.2. Intraductal papillary adenoma 3. Malignant epithelial neoplasms 3.1. Carcinoma In Situ * 3.2. Simple carcinomas 3.2.1. Tubular carcinoma 3.2.2. Tubulopapillary carcinoma 3.2.3. Solid carcinoma 3.2.4. Invasive micropapillary carcinoma 3.2.5. Comedocarcinoma 3.2.6. Anaplastic carcinoma 3.3. Non-simple carcinomas * 3.4. Ductal-associated carcinomas 3.4.1. Ductal carcinoma 3.4.2. Intraductal papillary carcinoma	4. Malignant epithelial neoplasms—special types 4.1. Squamous cell carcinoma 4.2. Adenosquamous carcinoma 4.3. Mucinous carcinoma 4.4. Lipid-rich carcinoma 4.5. Spindle cell carcinoma 4.6. Inflammatory mammary carcinoma 5. Malignant mesenchymal neoplasms of the mammary gland 5.1. Osteosarcoma 5.2. Chondrosarcoma 5.3. Fibrosarcoma 5.4. Hemangiosarcoma 5.5. Other sarcomas 6. Carcinosarcoma 7. Hyperplasia/Dysplasia of the teat 7.1. Hyperplasia of the teat 8. Neoplasms of the teat 8.1. Benign ductal-associated neoplasms 8.1.1. Ductal adenoma 8.1.2. Intraductal papillary adenoma 8.2. Malignant ductal-associated neoplasms 8.2.1. Ductal carcinoma 8.2.2. Intraductal papillary carcinoma 8.3. Carcinoma with epidermal infiltration (Paget-like disease)

* The use of these tumor types is discouraged.

**Table 4:** Histological grading methods used in FMT according to Elston and Ellis (1991) and Mills and colleagues (2015) [23,62].

	Score			
	0	1	2	3
Tubule formation				
EE, MMEE, REE	-	75%	10–75%	<10%
Nuclear pleomorphism				
EE, MMEE	-	Small, regular, uniform	Moderate variation in size and shape; vesicular nuclei; visible nucleoli	Marked variation in size and shape; bizarre and/or vesicular nuclei; prominent and/or multiple nucleoli
Mitotic count (in 10 HPF)				
EE ^a^	-	≤8	9–17	≥18
MMEE, REE	-	≤50	51–70	≥71
MGS	≤62	>62	-	-
Nuclear form				
REE	-	≤5% abnormal	6–25% abnormal	>25% abnormal
MGS	≤5%	>5%	-	-
Lymphovascular invasion				
REE, MGS	Absent	Present	-	-
	Grade			
	I	II		III
EE, MMEE	3–5	6–7		8–9
REE	3–5	6–7		8–10
MGS	0	1		2–3

EE—Elston and Ellis; MMEE—Mitotic Modified Elston and Ellis; REE—Revised Elston and Ellis; MGS—Mills Grading System; HPF—High-power field. ^a^ Mitotic count threshold adapted to a standard field diameter of 0.55 mm, HPF area of 0.237 mm^2^.

## Data Availability

No new data were created or analyzed in this study. Data sharing is not applicable to this article.
